# Analysis of Strategies for Managing Stress by Polish Nurses during the COVID-19 Pandemic

**DOI:** 10.3390/healthcare10102008

**Published:** 2022-10-12

**Authors:** Anna Stefanowicz-Bielska, Magdalena Słomion, Małgorzata Rąpała

**Affiliations:** 1 Division of Internal and Pediatric Nursing, Institute of Nursing and Midwifery, Faculty of Health Sciences with the Institute of Maritime and Tropical Medicine, Medical University of Gdansk, 80-211 Gdansk, Poland; 2 Department of Pediatric Surgery, Marciniak Hospital, 50-041 Wroclaw, Poland

**Keywords:** nurses, occupational stress, emotional stress, SARS-CoV-2, COVID-19

## Abstract

Background: The COVID-19 pandemic is having a negative impact on the mental health of nurses around the world. The aim of the study was to assess the sense of fear and the degree of exposure to SARS-CoV-2 infection and to estimate the influence of various factors on the fear of SARS-CoV-2. We analysed methods and strategies for coping with stress used by Polish nurses during the COVID-19 pandemic. Methods: The study was conducted using a diagnostic survey, which included a self-constructed questionnaire and a standardized psychological questionnaire: Brief-Cope. Results: A total of 361 nurses confirmed their participation in the study. Most of the nurses were ages 31 to 50 (48.2%), lived in a city (83.7%), and had a master’s degree in nursing (45.7%). Nurses ≥ 51 years of age, with ≥ 21 years of work experience and with secondary education in nursing and master of nursing were more likely than other nurses to agree with the statement, *I am afraid of contracting the SARS-CoV-2* (appropriately *p* = 0.009, *p* = 0.007, *p* = 0.014). During the outbreak of COVID-19, nurses most often took action to improve the situation, reflecting on and planning what to do. The most frequent means of coping with stress by Polish nurses during the COVID-19 outbreak were problem-focused strategies. The least frequent strategy was the use of substances (taking substances to alleviate unpleasant emotions), considered to be the least effective, but useful in some situations. Conclusions: Most nurses were afraid of being infected with COVID-19. The most frequently used strategies for coping with stress by Polish nurses during the COVID-19 pandemic were problem-focused strategies. Nurses should receive psychological support and assistance from the employer in improving their working conditions.

## 1. Introduction

COVID-19 is a highly contagious disease caused by the respiratory coronavirus SARS-CoV-2 [1]. In late 2019, the virus spread very quickly, affecting the health of many people around the world [2]. On 11 March 2020, the World Health Organization (WHO) declared a pandemic. The first case of SARS-CoV-2 infection in Poland was reported on 4 March 2020, in the western part of the country [3].

At the beginning of the COVID-19 pandemic, the Polish government took several drastic measures to protect the population. These measures introduced a number of different restrictions: a mandatory 14-day quarantine for people returning from abroad, remote work for administrative staff, suspension of classroom teaching in schools and universities, a limit to the number of people in shops at the same time, restrictions on the number of people congregating in churches, an obligation to cover the mouth and nose and the closure of certain services (i.e., hair salons, gyms) as well as cultural facilities (i.e., theatres, museums).

Limiting outdoor activities and regular exercises have affected most daily activities. Regular physical activity improves mental health reduces the risk of depression and improves the overall feelings of well-being [4].

COVID-19 has been particularly difficult for health care practitioners due to high contagiousness, unknowns regarding the virus and the disease, and the threat it poses to the lives of medical professionals [5].

Some hospitals were restructured. Many medical workers were assigned shifts in wards or hospitals for patients with COVID-19, and often these were extra shifts with longer hours. Healthcare professionals were at an increased risk of contracting the disease and transmitting it to other patients, coworkers and their family/friends. The health and safety of medical workers was essential to ensure the continuity and safety of care for the infected [6].

Nurses are the most numerous medical professionals in Poland and worldwide. While performing their tasks, nurses are exposed to many harmful, burdensome and dangerous physical elements as well as emotional and interpersonal stressors [7]. During their work, nurses often feel mental and physical fatigue, exhaustion, helplessness, skepticism and lack of joy in performing nursing activities as a result of constant tension at work [8]. They were the first to come into contact with infected patients during the COVID-19 outbreak. They play an important role in preventing the spread of infection, controlling the number of infections, and supporting patients in isolation [9]. They also play an important role in public education in preventing and reducing the spread of the disease.

The COVID-19 pandemic is having a negative impact on the mental health of nurses around the world. The COVID-19 pandemic significantly affects well-being, quality of working life and coping. Even though coping strategies are associated with both wellbeing and quality of working life, healthcare workers demonstrated an increase in negative coping strategies to deal with the escalation of work pressures [10]. Working with COVID-19 patients causes fear, anxiety, psychological distress, acute stress, post-traumatic stress, anger, depression, various degrees of mental crisis and burnout in healthcare professionals. Burnout has been linked to overall mental stress which could be work-related occupational hazard acquired when providing healthcare for patients. Burnout results from chronic stress in the workplace which has not been successfully managed [11,12].

The cognitive-behavioral therapy improves well-being and reduces perceived stress [13].

The aim of the study was to assess the sense of fear and the degree of exposure to SARS-CoV-2 infection and to exam the influence of various factors on the fear of SARS-CoV-2. We analysed the methods and strategies for coping with stress used by nurses during the COVID-19 pandemic.

## 2. Materials and Methods

### 2.1. Design

We conducted a cross-sectional study from 1 April to 30 June 2021 in a group of the Polish nurses based on own socio-demographic survey and the assessment of coping with stress ways and strategies.

### 2.2. Participants

The participants of the study were active nurses. A total of 21 selected health care entities, which have the largest number of the employed nurses, in various regions of Poland were invited to participate in the study, and 14 hospitals confirmed their participation.

The inclusion criteria were Polish nurses and their present activity in the job.

### 2.3. Methods

The study was conducted using a diagnostic survey, which included a self-constructed questionnaire about socio-demographic data and a standardized psychological questionnaire, Brief-Cope (Mini-COPE) created by Charles Carver (adapted to Polish conditions by Z. Juczyński and N. Ogińska-Bulik) [14,15,16].

The self-constructed questionnaire concerned the socio-demographic data of participants.

The first page of the questionnaire provided information about the study and an invitation to participate. The survey consisted of questions on socio-demographic data (gender, age, place of residence, education, place of work, years of employment in the profession). It assessed the nurses’ level of fear of being infected with SARS-CoV-2 (*I’m afraid of contracting SARS-CoV-2 virus:* strongly agree, agree, disagree, strongly disagree); the degree of perceived risk of SARS-CoV-2 infection: (*I am at a higher risk of contracting COVID-19 disease than anyone else*: strongly agree, agree, disagree, strongly disagree); and the possibility of contact with a patient infected with SARS-CoV-2 (*During my work, I took care of a patient infected with the SARS-CoV-2 virus*: yes, no).

#### The Brief-Cope Questionnaire

The Brief-Cope Questionnaire is used to assess ways of coping with stress. It consists of 28 statements included in 14 strategies for coping with stress: active coping, planning, use of instrumental support, use of emotional support, turning to religion, positive reframing and development, behavioural disengagement, acceptance, venting, denial, self-distraction, self-blame, substance use, and humour. There are two statements for each strategy. The respondent answers on a 4-point scale from 0 to 3, where 0 means: I hardly ever do this, and 3 means: I almost always do this. The higher the score, the more frequently the respondent uses the given strategy [16].

These subscales of the COPE can be grouped into problem-focussed (active coping, planning, use of instrumental support), emotion-focussed (positive reframing, use of emotional support, acceptance, sense of humour, turning to religion) and dysfunctional coping (self-distraction, denial, venting, substance use, behavioural disengagement, self-blame) [17,18].

The electronic version of the Brief-Cope Questionnaire (Mini-COPE) was used with permission from the Psychological Test Laboratory of the Polish Psychological Association [15].

### 2.4. Data Collection

The data was collected using an online psychological survey and questionnaire created in a Google form. A link to the survey and to the questionnaire was sent to all nurses via emails sent out by directors of selected health care facilities. Participation in the study was voluntary. The study was approved by the individual health facilities and accepted by the Independent Bioethics Committee for Scientific Research at the Medical University of Gdańsk (NKBBN/365/2021).

### 2.5. Statistical Methods

For each parameter, mean (X), median (M), standard deviation (SD, range/min, max), lower and upper quartile (25Q, 75Q) were calculated. Statistical significance between means for different groups was calculated by one-way analysis of variance (ANOVA), alternatively using the nonparametric U Mann-Whitney test (for two groups) or Kruskal-Wallis test (for more than two groups), when the variances in groups were not homogeneous (the homogeneity of variance was determined by the Levene’s test).

Statistical significance between frequencies was calculated by the chi-square test χ^2^_df_ with corresponding degree of freedom df (df = (m − 1) × (n − 1), where m is number of rows and n is number of columns).

A *p* value of less than 0.05 was required to reject the null hypothesis. Statistical analysis was performed using EPIINFO Ver. 7.2.3.1 (Epi Info™ is a trademark of the Centers for Disease Control and Prevention (CDC). 1600 Clifton Road Atlanta, GA 30329-4027 USA) and Statistica Ver. 13.3.(TIBCO Sofware Inc. 3307 Hillview Avenue Palo Alto, CA 94304 USA) software packages.

## 3. Results

### 3.1. Characteristics of the Participants

The link to the survey and the psychological questionnaire was sent to 6560 nurses: 361 (5.5%) nurses confirmed their participation in the study, including 19 men and 342 women. A total of 6199 nurses refused. Most of the nurses were ages 31 to 50 (174/361, 48.2%), lived in a city (302/361, 83.7%), and had a master’s degree in nursing (165/361, 45.7%). The nurses worked in outpatient medical clinics (63/361, 17.5%), in paediatric wards (61/361, 16.9%), in surgical wards (57/361, 15.8%), in internal diseases wards (48/361, 13.3%), in surgical units (33/361, 9.1%), in intensive care units for adults (28/361, 7.8%), in emergency departments (21/361, 5.8%), in intensive care units for children (13/361, 3.6%), in hospices (13/361, 3.6%), in psychiatric wards for adults (11, 3.1%), in medical universities (7/361, 1.9%), in health care and curative institutions (5/361, 1.4%) and in psychiatric wards for children (1/361, 0.2%).

The study participants were divided into groups of nurses working in clinics (63/361, 17.5%), in hospital wards (273/361, 75.6%) in hospices and health care and curative institutions (18/361, 5%), and in medical universities (7/361, 1.9%). Hospital nurses were divided into nurses working in paediatric wards (61/361, 22.3%), internal diseases wards (48/361, 17.6%), surgical units and surgical wards (90/361, 33%), intensive care units (41/361, 15%), emergency departments (21/361, 7.7%) and psychiatric wards for adults and for children. (12/361, 4.4%). The majority of participants (61.5%, 222/361) had ≥ 21 years of employment in the profession (Table 1).

### 3.2. The Level of Fear and the Degree of Exposure to SARS-CoV-2 Infection of the Surveyed Nurses

The survey addressed issues of attitudes towards virus infection in the time of the COVID-19 pandemic. Most nurses (286/361, 79.3%) declared being afraid of SARS-CoV-2 infection, and 276/361, 76.5% nurses thought they were more likely to contract COVID-19 than anyone else. Most nurses estimated that they had a high level of knowledge about COVID-19 and about infection prevention and treating an infected patient (50.7%), 47.9% had mid-level knowledge, and 1.4% had low-level knowledge. Most nurses (74.8%, 270/361) had direct contact with a patient infected with SARS-CoV-2.

### 3.3. The Influence of Various Factors on the Fear of Being Infected with SARS-CoV-2

Nurses ≥ 51 years of age, with ≥ 21 years of work experience and with secondary education in nursing and master of nursing agreed with the statement *I am afraid of contracting the SARS-CoV-2 virus* much more often than other groups. Gender, place of residence, place of work and contact with a patient infected with SARS-CoV-2 did not affect the fear of being infected with SARS-CoV-2 (Table 2). The nurses’ self-assessment of their knowledge of COVID-19 and prevent infection and deal with an infected patient did not depend on their level of fear of contracting the SARS-CoV-2 (*p* = 0.236) or contact with a patient infected with SARS-CoV-2 (*p* = 0.636). Most nurses (212/270, 78.5%) who had contact with a patient infected with SARS-CoV-2 stated that they were afraid of being infected.

### 3.4. Ways and Strategies of Coping with Stress

Nurses’ responses to individual statements in the Brief-Cope questionnaire were analysed. Most nurses responded that they have often or almost always been concentrating their efforts on doing something about the situation they’re in (45% and 42%, respectively) and they been taking action to try to make the situation better (37% and 51%, respectively). The majority of nurses stated that they never take the following action: I’ve been saying to myself “this isn’t real (48%), I’ve been using alcohol or other substances to make myself feel better (72%), I’ve been using alcohol or other drugs to help me get through it (75%), I’ve given up the attempt to cope (51%) (Table 3).

**Figure 1:**
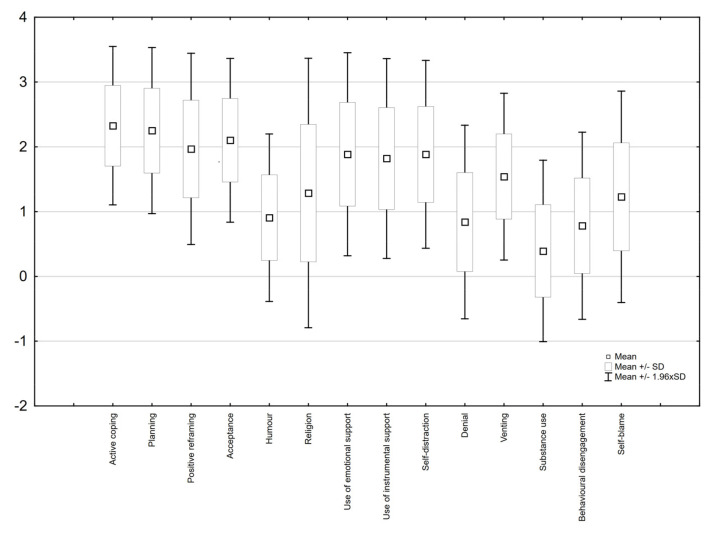
Strategies for coping with stress.

Additionally, 14 strategies of coping with stress were analysed in Figure 1. The highest average number of points was earned by the nurses for strategies: active coping (2.33 ± 0.62) and planning (2.25 ± 0.65); the lowest average number of points was earned by the nurses for strategies: substance use (taking substances to alleviate unpleasant emotions—0.39 ± 0.71—hardy ever and rarely).

### 3.5. Influence of Various Factors on Strategies of Coping with Stress

The influence of gender, age, place of residence, place of work, years of employment in the profession and education on various strategies of coping with stress was analysed (Table 4).

For the purposes of the analysis, three groups of strategies for coping with stress were combined: problem-focused, emotion-focused and dysfunctional coping. The workplace has been shown to influence emotion-centered strategies. People working in hospices and nursing homes use emotional strategies to deal with stress. It has been shown that people with a master’s degree mainly use problem-focused strategies compared to other people with a different level of education. Years of work have an impact on the use of strategies based on the dysfunctional coping strategy. People who have worked for ≤ 10 years are more likely to use the dysfunctional coping strategy. However, the influence of gender, age and place of permanent residence was not found.

Group 1—Outpatient medical clinics, Group 2—Hospital wards (paediatric ward, surgical ward, internal diseases ward, surgical unit, intensive care unit for adults, emergency department, intensive care unit for children, psychiatric ward for adults, psychiatric ward for children, Group 3—Hospice, health care and curative institutions, Group 4—Medical University

## 4. Discussion

Nurses are one of medical professional groups at risk of being under constant stress [19]. Before the COVID-19 pandemic, the stress among Polish nurses was caused by responsibility for human health and life, high workload, shift work, low salaries, insufficient number of employees, poor organization of work, conflicts on the therapeutic team, inappropriate interpersonal relationships, lack of support, contact with gravely ill and dying patients and their families [20,21].

It is well known that constant and permanent stress may lead to occupational burnout.

Bordoagni et al. [22] evaluated the attachment style and mentalization capacity in nurse professionals and nursing students and investigated the impact of these factors on burnout in professional nurses. The author’s results importantly highlight the usefulness of the assessment of attachment style and mentalization in professional nurses to elucidate and reinforce the role of protective factors against burnout [22].

Safiye et al. [23] showed that being a woman and working on the COVID-19 frontline implies a higher burnout, while the level of burnout decreases with better socioeconomic status and more children. Resilience, capacity for mentalizing, and burnout syndrome among healthcare workers are interrelated phenomena, which have important professional implications [23].

The COVID-19 pandemic is a huge challenge for active nurses. A significant additional stressor fear of contracting the virus and developing the COVID-19 infection was added to an already extensive list of stressors. The COVID-19 pandemic significantly worsened working conditions associated with greater workload.

The problem of stress and coping strategies among nurses has been raised by various researchers.

Isa et al. [24] conducted a cross-sectional study with a group of 85 nurses working in the emergency and intensive care units of the largest hospital in Brunei. The authors observed that nurses in the emergency department and critical care units were mostly using the planful problem-solving coping strategy, followed by positive reappraisal, distancing, seeking social support and accepting responsibility strategies. Confrontative coping and escape-avoidance behaviours were the least exhibited strategies. In addition, those who worked in the medical intensive care unit scored significantly higher on escape-avoidance coping behaviours compared with those working in the emergency department [24].

In contrast, in our study the nurses who work in hospice, healthcare and curative institutions use emotional—focused strategies.

Chinese researchers assessed the moderating effect of coping strategies on the relationship between work stress and job performance in 852 nurses. They found that positive coping strategies reduced or mitigated the negative effects of work stress on job performance, and that negative coping strategies increased the negative effects of work stress [25].

Tesfaye et al. [26] described strategies for coping with work stress among 433 nurses working in public hospitals. The most preferred strategies were social support and full planned problem-solving. The strategy of coping with flight and avoidance was the least used [26].

In contrast, in our study the most preferred strategies were active and planning. The least used strategy was substance use (taking substances to alleviate unpleasant emotions).

A Polish cross-sectional study by Betke et al. [20] included 91 nurses from central Poland. Nurses from the study group most often declared the use of strategies included in the active ways of coping with stress and focused on the problem [20].

In our study the nurses declared the most often emotion-focused strategies for coping with stress.

Kowalczuk et al. [27] researched the relationship between excessive sleepiness and insomnia in interaction with selected socio-professional factors and coping strategies among 448 nurses working in hospitals of Podlaskie Voivodeship in Poland. The most commonly used coping strategies were active strategies (active coping, planning). Avoidance strategies (behavioural disengagement, substance use) were the least frequently used. Nurses with higher levels of education and sleep problems used the strategies of humour, behavioural disengagement, substance use and religion less frequently than those with lower levels of education. Nurses working in emergency wards experiencing excessive sleepiness used the strategies of humour, religion and positive reframing less often than those working in other wards, while those suffering from insomnia used the strategy of humour more often than those working in other wards [27].

The assessment of stress levels in nurses and the analysis of coping strategies by nurses during the COVID-19 pandemic is an interesting issue.

A Turkish study by Subas et al. [28] included 337 nurses, 39 midwives, 16 doctors and 50 other medical professionals. The authors of the study showed that seeking social support was the most common method of coping with stress by health care workers during the COVID-19 pandemic [28].

Ziarko et al. [29] assessed the level of stress among doctors (n = 41), nurses (n = 114) and paramedics (n = 15) during the COVID-19 pandemic and analysed whether there was a relationship between the stress level and mental health of healthcare workers in the context of coping with stress. Among medical workers, nurses experienced the most severe stress. The increasing level of stress was accompanied by insomnia and depression. Maladaptive methods of coping with stress (e.g., the use of psychoactive substances) resulted in the deterioration of mental health in the study group. The habitual use of maladaptive strategies may, in the short term, bring relief in the form of reducing the negative consequences of stress and facilitate mobilization or maintain productivity at work [29].

In our study use of substances (taking substances to alleviate unpleasant emotions) was very rare.

Another Polish study by Puto et al. [30] involved 151 nurses working with patients infected with SARS-CoV-2 and patients who were not infected during the COVID-19 pandemic. Nurses working with patients who had SARS-CoV-2 experienced more stress than those working with uninfected patients. Nurses working with patients who had SARS-CoV-2 coped with stress using strategies focused on the problem (active coping, seeking instrumental support) and on emotions (seeking encouragement, understanding and support from other people). On the other hand, nurses working with uninfected patients most often chose strategies focused solely on the problem (active coping and planning) [30].

The rapid spread of COVID-19 infection was connected with restrictions of normal life and difficulties with job of health care workers. Stress, anxiety and depression can be fueled by uncertainty and disappointment of being unable to perform in the competitions as planned. Some nurses perceive the pandemic COVID-19 as a traumatic experience because takes a physical and emotional toll on everyone [31].

Sut Txi et al. [31] found among athletes during COVID-19 period that the depression, anxiety and stress scores in the current sample group were lower than those from other groups of people [31].

This conclusion is consistent with the statement that regular sports participation decreases the risk of emotional distress such as depression, anxiety and stress [31]. It should be helpful for healthcare workers.

In summary, our research showed that almost 80% of nurses were afraid of being infected with SARS-CoV-2, and 77% of those surveyed believe they were more likely to get COVID-19 than anyone else. Older nurses, with longer work experience and with secondary education, more often agreed with the statement, *I am afraid of contracting SARS-CoV-2*. The most common strategies for coping with stress were active coping, planning and acceptance. The most rarely used strategy was the use of psychoactive substances. It is worth emphasizing that active coping with stress and planning are treated as problem-focused strategies, while acceptance is considered as an adaptive strategy in situations where active coping with stress is effective [16,17].

It is worth noting that an interesting issue would be to conduct a study comparing strategies for coping with stress among nurses before and during the COVID-19 pandemic. However, the authors of the study were not able to predict that there would be an additional stressor in the form of the COVID-19 pandemic.

### Study Limitations

The main limitation of the study was the fact that only 5% of nurses invited to the study responded to the survey. The second limitation of the study is that it was conducted electronically as the authors of the study were unable to recruit participants for the study in person due to the COVID-19 pandemic. The authors of the study used only one psychometric questionnaire. They did not assess the participants’ level of anxiety, stress, psychological well-being and psychological health, or factors affecting lifestyles in the respondents. The authors of the article will expand their research in future.

## 5. Conclusions

Most nurses were afraid of being infected with COVID-19. The most frequently used strategies for coping with stress by Polish nurses during the COVID-19 pandemic were problem-focused strategies. The least frequently used strategy was the use of psychoactive substances, which were considered to be the least effective but useful in some situations. Nurses should receive psychological support and assistance from the employer in improving their working conditions.

## Figures and Tables

**Table 1:** Characteristics of the participants.

Socio-Demographic Data		N (%)
Gender	F	342 (94.7%)
	M	19 (5.3%)
Age (years)	21–30	57 (15.8%)
	31–50	174 (48.2%)
	≥51	130 (36%)
Place of residence	City	302 (83.7%)
	Village	59 (16.3%)
Education	Master of nursing	165 (45.7%)
	Bachelor of nursing	103 (28.5%)
	Secondary education in nursing	93 (25.8%)
Place of work	Outpatient medical clinic	63 (17.5%)
	Paediatric ward	61 (16.9%)
	Surgical ward	57 (15.8%)
	Internal diseases wards	48 (13.3%)
	Surgical units	33 (9.1%)
	Intensive care units for adults	28 (7.8%)
	Emergency departments	21 (5.8%)
	Intensive care units for children	13 (3.6%)
	Hospices	13 (3.6%)
	Psychiatric wards for adults	11 (3.1%)
	Medical universities	7 (1.9%)
	Health care and curative institution	5 (1.4%)
	Psychiatric wards for children and adults	1 (0.2%)
Years of employment in the profession (years)	≤10	89 (24.6%)
	11–20	50 (13.9%)
	≥21	222 (61.5%)

**Table 2:** Influence of various factors on fear of SARS-CoV-2 infection.

Factors		I Am Afraid of Contracting the SARS-CoV-2		
		Agree	Disagree	*p*
Gender	F	273 (79.8%)	69 (20.2%)	0.233
	M	13 (68.4%)	6 (31.6%)	
Age (years)	21–30	38 (66.7%)	19 (33.3%)	0.009
	31–50	136 (78.2%)	38 (21.8%)	
	≥51	112 (86.2%)	18 13.9%)	
Place of residence	City	239 (79.1%)	63 (20.9%)	0.928
	Village	47 (79.7%)	12 (20.4%)	
Education	Master of nursing	134 (81.2%)	31 (18.8%)	0.014
	Bachelor of nursing	72 (69.9%)	31 (30.1%)	
	Secondary education in nursing	80 (86%)	13 (14%)	
Place of work	Outpatient medical clinics	52 (82.5%)	11 (17.5%)	0.072
	Hospital wards (paediatric ward, surgical ward, internal diseases ward, surgical unit, intensive care unit for adults, emergency department, intensive care unit for children, psychiatric ward for adults, psychiatric ward for children	219 (80.2%)	54 (19.8%)	
	Hospice, health care and curative institutions	10 (55.6%)	8 (44.4%)	
	Medical university	5 (71.4%)	2 (28.6%)	
Years of employment in the profession	≤10	61 (68.5%)	28 (31.5%)	0.007
	11–20	38 (76.0%)	12 (24.0%)	
	≥21	187 (84.2%)	35 (15.8%)	
Caring for a patient infected with SARS-CoV-2	Yes	212 (78.5%)	58 (21.5%)	0.569
	No	74 (81.3%)	17 (18.7%)	

**Table 3:** Nurses’ responses to statements in the Brief-Cope questionnaire.

Statement	Frequency of Activities			
	I Have Hardly ever Done This	I Have Been Doing This a Bit	I Have Been Doing This a Medium Amount	I Have Been Doing This a Lot
I’ve been turning to work or other activities to take my mind off things.	27 (7.5%)	63 (17.5%)	162 (44.9%)	109 (30.2%)
I’ve been concentrating my efforts on doing something about the situation I’m in.	6 (1.7%)	38 (10.5%)	164 (45.4%)	153 (42.4%)
I’ve been saying to myself “this isn’t real”.	173 (47.9%)	127 (35.2%)	43 (11.9%)	18 (5%)
I’ve been using alcohol or other substances to make myself feel better.	259 (71.8%)	67 (18.6%)	24 (6.7%)	11 (3.1%)
I’ve been getting emotional support from others.	33 (9.1%)	105 (29.1%)	123 (34.1%)	100 (27.7%)
I’ve given up trying to deal with it.	155 (42.9%)	121 (33.5%)	72 (19.9%)	13 (3.6%)
I’ve been taking action to try to make the situation better.	8 (2.2%)	35 (9.7%)	135 (37.4%)	183 (50.7%)
I’ve been refusing to believe that it has happened.	138 (38.2%)	130 (36%)	70 (19.4%)	23 (6.4%)
I’ve been saying things to let my unpleasant feelings escape.	56 (15.5%)	122 (33.8%)	121 (33.5%)	62 (17.2%)
I’ve been getting help and advice from other people.	33 (9.14%)	76 (21.1%)	156 (43.2%)	96 (26.6%)
I’ve been using alcohol or other drugs to help me get through it.	272 (75.4%)	53 (14.7%)	26 (7.2%)	10 (2.8%)
I’ve been trying to see it in a different light, to make it seem more positive.	19 (5.3%)	75 (20.8%)	165 (45.7%)	102 (28.3%)
I’ve been criticizing myself.	72 (19.9%)	145 (40.2%)	103 (28.5%)	41 (11.4%)
I’ve been trying to come up with a strategy about what to do.	9 (2.5%)	51 (14.1%)	163 (45.2%)	138 (38.2%)
I’ve been getting comfort and understanding from someone.	15 (4.2%)	89 (24.7%)	150 (41.6%)	107 (29.6%)
I’ve given up the attempt to cope.	184 (51%)	111 (30.8%)	49 (13.6%)	17 (4.7%)
I’ve been looking for something good in what is happening.	28 (7.8%)	69 (19.1%)	152 (42.1%)	112 (31%)
I’ve been making jokes about it.	66 (18.3%)	112 (31.0%)	137 (38%)	46 (12.7%)
I’ve been doing something to think about it less, such as going to movies, watching TV, reading, daydreaming, sleeping, or shopping.	34 (9.4%)	93 (25.8%)	149 (41.3%)	85 (23.6%)
I’ve been accepting the reality of the fact that it has happened.	13 (3.6%)	50 (13.9%)	186 (51.5%)	112 (31%)
I’ve been expressing my negative feelings.	42 (11.6%)	132 (36.6%)	131 (36.3%)	56 (15.5%)
I’ve been trying to find comfort in my religion or spiritual beliefs.	119 (33%)	87 (24.1%)	79 (21.9%)	76 (21.1%)
I’ve been trying to get advice or help from other people about what to do.	29 (8%)	105 (29.1%)	148 (41%)	79 (21.9%)
I’ve been learning to live with it.	9 (2.5%)	54 (15%)	189 (52.4%)	109 (30.2%)
I’ve been thinking hard about what steps to take.	8 (2.2%)	34 (9.4%)	157 (43.5%)	162 (44.9%)
I’ve been blaming myself for things that happened.	105 (29.1%)	135 (37.4%)	85 (23.6%)	36 (10%)
I’ve been praying or meditating,	121 (33.5%)	84 (23.3%)	96 (26.6%)	60 (16.6%)
I’ve been making fun of the situation.	268 (74.2%)	65 (18%)	19 (5.3%)	9 (2.5%)

**Table 4:** Influence of various factors on strategies of coping with stress.

Factors		Strategies for Coping with Stress			
		N	Problem -Focused Strategies	Emotion -Focused Strategies	Dysfunctional -Coping Strategies
Gender	F	342	6.5 (5.5 ÷ 7.5)	8.0 (7.0 ÷ 10.0)	6.5 (5.0 ÷ 8.0)
	M	19	6.0 (6.0 ÷ 8.0)	8.5 (7.0 ÷ 10.0)	6.5 (4.5 ÷ 7.5)
*p*			0.943	0.619	0.794
Age (years)	21–30	57	6.5 (6.0 ÷ 7.5)	8.0 (6.5 ÷ 9.5)	7.0 (6.0 ÷ 8.5)
	31–50	174	6.5 (5.5 ÷ 7.5)	8.0 (6.5 ÷ 10.0)	6.5 (5.0 ÷ 8.0)
	≥51	130	6.5 (5.5 ÷ 7.5)	8.0 (7.0 ÷ 9.5)	6.5 (4.5 ÷ 8.0)
*p*			0.998	0.857	0.097
Place of residence	City	302	6.5 (5.5 ÷ 7.5)	8.0 (6.5 ÷ 10.0)	7.0 (5.0 ÷ 8.0)
	Village	59	6.5 (5.5 ÷ 7.5)	8.0 (7.0 ÷ 9.0)	6.5 (4.5 ÷ 7.0)
*p*			0.868	0.941	0.177
Education	Master of nursing	165	7.0 (6.0 ÷ 8.0)	8.0 (7.0 ÷ 10.0)	6.5 (4.5 ÷ 8.0)
	Bachelor of nursing	103	6.0 (5.5 ÷ 7.0)	8.0 (6.5 ÷ 9.5)	7.0 (5.0 ÷ 8.5)
	Secondary education in nursing	93	6.5 (5.0 ÷ 7.5)	8.0 (6.5 ÷ 9.5)	7.0 (5.5 ÷ 8.0)
*p*			0.003	0.379	0.160
Years of employment in the occupation	≤10	89	6.5 (6.0 ÷ 7.5)	8.0 (6.5 ÷ 10.0)	7.5 (6.0 ÷ 9.0)
	11–20	50	6.75 (5.5 ÷ 8.0)	8.0 (6.5 ÷ 10.0)	6.5 (5.5 ÷ 8.0)
	≥21	222	6.5 (5.5 ÷ 7.5)	8.0 (6.5 ÷ 10.0)	6.5 (4.5 ÷ 8.0)
*p*			0.345	0.996	0.002
Place of work	1	63	6.5 (5.5 ÷ 7.5)	7.5 (6.5 ÷ 10.0)	7.0 (5.5 ÷ 8.5)
	2	273	6.5 (5.5 ÷ 7.5)	8.0 (6.5 ÷ 9.5)	6.5 (4.5 ÷ 8.0)
	3	18	7.25 (6.0 ÷ 8.0)	9.5 (8.0 ÷ 11.5)	6.5 (5.0 ÷ 9.0)
	4	7	6.5 (6.0 ÷ 8.0)	7.0 (6.0 ÷ 7.5)	6.0 (4.5 ÷ 8.0)
*p*			0.245	0.030	0.506

## Data Availability

Data available on request due to privacy restriction.
